# Long non-coding RNA H19 contributes to spinal cord ischemia/reperfusion injury through increasing neuronal pyroptosis by miR-181a-5p/HMGB1 axis

**DOI:** 10.18632/aging.204160

**Published:** 2022-07-05

**Authors:** Lili Guo, Dan Wang, Hildrich Yasmal Alexander, Xiaoyan Ren, Hong Ma

**Affiliations:** 1 Department of Anesthesiology, First Hospital of China Medical University, Shenyang 110001, Liaoning Province, China

**Keywords:** spinal cord ischemia-reperfusion injury, H19, miR-181a-5p, HMGB1, pyroptosis

## Abstract

Pyroptosis, a programmed inflammatory necrotizing cell death, is likely involved in spinal cord ischemia-reperfusion (SCI/R) injury, but the mechanisms initiating driving neuronal pyroptosis must be further revealed. The aim of this study is to unravel the mechanism of long non-coding RNA (lncRNA) H19 during SCI/R. SCI/R model was induced in C57BL/6 mice by blocking the aortic arch *in vivo*, and oxygen-glucose deprivation/reperfusion (OGD/R) injury model of PC12 cells was established *in vitro*. Our results showed that H19 and HMGB1 expression was upregulated, while miR-181a-5p was downregulated in the SCI/R mice and OGD/R-treated PC12 cells. SCI/R induced pathological damage, pyroptosis and inflammation compared with the sham group. H19 acted as a molecular sponge to suppress miR-181a-5p, and HMGB1 was identified as a direct target of miR-181a-5p. MiR-181a-5p overexpression inhibited the increase of IL-1β, IL-18 and TNF-α production and NLRP3, ASC, and Cleaved-caspase-1 expression in OGD/R-treated PC12 cells; while miR-181a-5p silencing exerted opposite effects. HMGB1 overexpression reversed H19 knockdown-mediated the inhibition of pyroptosis and inflammation in OGD/R-treated PC12 cells. *In vivo*, H19 knockdown promoted the hind limb motor function recovery and alleviated the pathological damage, pyroptosis and inflammation induced by SCI/R. LncRNA H19/miR-181a-5p/HMGB1 pathway contributes to pyroptosis via activating caspase1 signaling during SCI/R, suggesting that this axis may be a potent therapeutic target in SCI/R.

## INTRODUCTION

Spinal cord ischemia-reperfusion (SCI/R) injury a common serious complicating disease (5%-10%) post thoracic and abdominal aortic aneurysm surgery [1], is also an important prognostic factor for the patients [2]. SCI/R injury could cause inflammatory response, nervous system injury, eventually leading to paralysis and paraplegia. Currently, there are many measures to improve SCI/R-induced damage, including shunt surgery, drainage surgery and medication [3–5], but with rarely satisfactory results. Therefore, it is important to unravel the mechanism of SCI/R to find an effective treatment.

Accumulating evidence revealed that nerve cells have a large demand for energy and are prone to ischemic injury in SCI/R injury [6]. Pyroptosis is one of the causes of nerve cell death which is conditioned by gasdermin D (GSDMD) [6, 7]. During SCI/R injury, inflammasome, represented by NLRP3, activates caspase-1 to cleave GSDMD and induce production of IL-1β and IL-18, leading to pyroptosis [8]. Li et al. uncovered that interference with the absent in melanoma 2 (AIM2) gene attenuates SCI/R-induced pyroptosis of spinal cells by inhibiting inflammasome activation and production of cleaved caspase-1 and IL-1β [9].

Accumulating evidence showed that lncRNAs are promising regulators of cell death and inflammation during SCI/R. For example, Jia et al. demonstrated that silencing of lncRNA TUG1 alleviated inflammatory damage post SCI/R by repressing miR-29b-1-5p and the NF-κB/IL-1β signaling [10]. Liu et al. revealed that lncRNA CasC7 expression was downregulated in SCI/R rats and oxygen-glucose deprivation/reperfusion (OGD/R)-induced SH5Y-SY neurons, and upregulation of CasC7 by NaSH preprocessing contributed to protect spinal cord via increasing miR-30c expression [11]. LncRNA H19 is correlated with neurological disorders such as intracranial aneurysms, ischemic stroke, glioma, and neuroblastoma [12]. Recent studies indicate that abnormal lncRNA H19 expression affected neurons apoptosis and inflammation after SCI [13], and lncRNA H19 was reported to regulate microglia pyroptosis [14, 15]. However, the exact role of H19 in neuron pyroptosis during SCI/R is still not well understood.

One of the most classic action modes of lncRNAs is so called ceRNA, in which they share same miRNAs with specific mRNAs. It was reported that miR-181a was repressed in N-methyl-4-phenylpyridinium (MPP+)-induced neuroblastoma cells, which inhibited autophagic apoptosis, thereby contributing to Parkinson’s disease (PD) [16]. A recent study reported that miR-181a-5p was also repressed in OGD-managed human cardiomyocytes and ischemia-reperfusion administrated mice, and inhibited cell apoptosis and inflammation [17]. Moreover, miR-181a-5p inhibits myocardial inflammation and oxidative stress [18]. Nevertheless, the detailed function of miR-181a-5p in SCI/R-induced spinal neurons were largely unknown.

Herein, the action mechanism of lncRNA H19/miR-181a-5p pathway is to be investigated in OGD/R-induced PC12 cells and SCI/R mice models *in vivo*. Taken together, H19 silence alleviated SCI/R injury through inhibiting neuronal pyroptosis by miR-181a-5p upregulation.

## MATERIALS AND METHODS

### Ethics statement

The study was approved by the Ethics Committee of the Center of First Hospital of China Medical University (Shenyang, China).

### Construction of the experimental SCI/R model

C57BL/6 mice (12-15 weeks) were supplied by the Animal Center of First Hospital of China Medical University. The cages were set in a clean animal room at 22-24° C, relative humidity of ~55% and 12/12-h light/dark cycle. Mice were divided into experimental groups and the sham group (n = 10). A model of SCI/R was prepared as previously described [9]. The mice in the sham group underwent the same procedure without clamping.

### Hematoxylin-eosin staining

The L4-6 segments were collected and fixed with 4% paraformaldehyde. Embedded with paraffin wax, the samples were sectioned into10-μm thin slices. for hematoxylin-eosin staining. The sections were dewaxed, dehydrated, and then stained with hematoxylin for 5 minutes and eosin for 2 minutes. After permeabilization, they were observed with a microscope (Olympus, Japan).

### Basso mouse scale

The Basso mouse scale (BMS) was used to evaluate the functions of SCI/R mice as described previously [19], at days -1 (before SCI/R surgery), 0 1 and 7.

### Assessment of Evans blue extravasation

Evans blue (EB) fluorescence was used to assess blood spinal cord barrier (BSCB) leakage as previously described [20].

### Cell culture and treatment

The PC12 nerve cell line was cultured in a 37° C incubator with 5% CO_2_ in Dulbecco’s modified Eagle’s medium (DMEM, Gibco, Carlsbad, CA, USA) containing 10% fetal bovine serum. For OGD treatment, cells were cultured in DMEM without glucose and placed in a hypoxic chamber at 37° C with 95% N_2_ and 5% CO_2_ for 2 h. Then, cells were kept in an atmosphere with 95% air and 5% CO_2_ for 12 h, and medium was renewed with normal DMEM. The control cells were cultured under normal conditions.

Lipofectamine 2000 (Invitrogen, Inc., Carlsbad, CA, USA) was used for the cell transfection, when the density reached 80% confluence, following the reagent’s instructions. H19 shRNAs, miR-181a-5p mimics, miR-181a-5p inhibitor and corresponding negative controls were obtained from GenePharma (Shanghai, China).

### RNA isolation and RT-qPCR

Total RNA was isolated with the TRIzol reagent (Thermo Fisher Scientific) and reversely transcribed using a PrimeScript RT Kit (TaKaRa, Dalian, China). Quantitative PCR reactions were conducted with SYBR Premix Ex Taq II (Thermo Fisher Scientific) according to Kit’s instructions. U6 and GAPDH were respectively used as loading controls for miRNAs and long transcripts, and relative abundance of the RNAs was computed with the 2^–ΔΔCt^ method.

### Western blot assay

Total protein was drawn, and equal amounts of protein were segregated by 10% SDS-PAGE and transferred to a PVDF membrane (Millipore, Bedford, MA, USA), which was blocked by 5% skim milk for 1 h at RT. The PVDF membranes were subsequently incubated with primary antibodies (all purchased from Abcam, Waltham MA, USA) overnight at 4° C: anti-ASC (1:350), anti-NLRP3 (1:750), caspase-1 (1:600) and GAPDH (loading control, 1:800), and then washed and incubated with a peroxidase-conjugated IgG (1:1500, Millipore) for 2 h at RT. Bands of visualized with Enhanced Chemiluminescence Kit (Amersham Pharmacia Biotech) under a ChemiDoc XRS Imaging System (Bio-Rad, Hercules, CA, USA). The abundance of the band was quantified by Image J software (National Institutes of Health).

### Nissl staining

Nissl staining was used to assess neuron survival according to a previous citation [21].

### Enzyme linked immunosorbent assays (ELISA)

The levels of IL-1β (RLB00), TNF-α (RTA00), and IL-18 (EK0592) were quantified on a microplate reader by ELISA kits according to the manufacturer’s instructions.

### Apoptosis detection assay

A terminal deoxyribonucleotidyl transferase-mediated dUTP-digoxigenin nick-end labeling (TUNEL) assay kit (Abcam) was used to detect the apoptotic cells.

### RNA pull-down assay

The bio-probe-NC, bio-miR-181a-5p-WT and bio-miR-181a-5p-MUT were synthesized by the Ribobio (Guangzhou, China). The interaction between miR-181a-5p and H19 in PC12 cells was confirmed by RNA pull-down assay according to the approach described previously [22].

### RNA immunoprecipitation (RIP)

The interaction between miR-181a-5p and HMGB1 was verified by RIP based on Argonaute 2 (Ago2) antibody according to the protocol of Magna RIP Kit (Millipore) [23].

### Dual luciferase reporter gene assay

Luciferase reporter gene constructs were established according to the approach described formerly [24]. Cells were co-transfected with wild-type (WT-H19 or WT-HMGB1) or mutant (MUT-H19 or MUT-HMGB1) constructs and oligos (miR-181a-5p mimics, miR-181a-5p inhibitor or corresponding controls) using lipofectamine 3000 transfection reagent (Thermo Fisher Scientific). Dual luciferase assay was performed 24 h post-transfection using the dual luciferase reporter assay system (Promega, Madison, WI, USA) according to the manufacturer’s instructions.

### Statistical analysis

Data analysis was conducted by SPSS version 22.0 software (IBM SPSS. Armonk, NY, USA). All data were presented as the mean ± standard deviation (SD) from three independent experimental repeats. Student’s T-test was used for the comparison between two groups, and one-way analysis of variance (ANOVA) was performed for comparison among multiple groups followed by Tukey’s test. P<0.05 was considered as statistically significant difference.

## RESULTS

### SCI/R induced pyroptosis and promoted lncRNA H19 expression level

**Figure 1:**
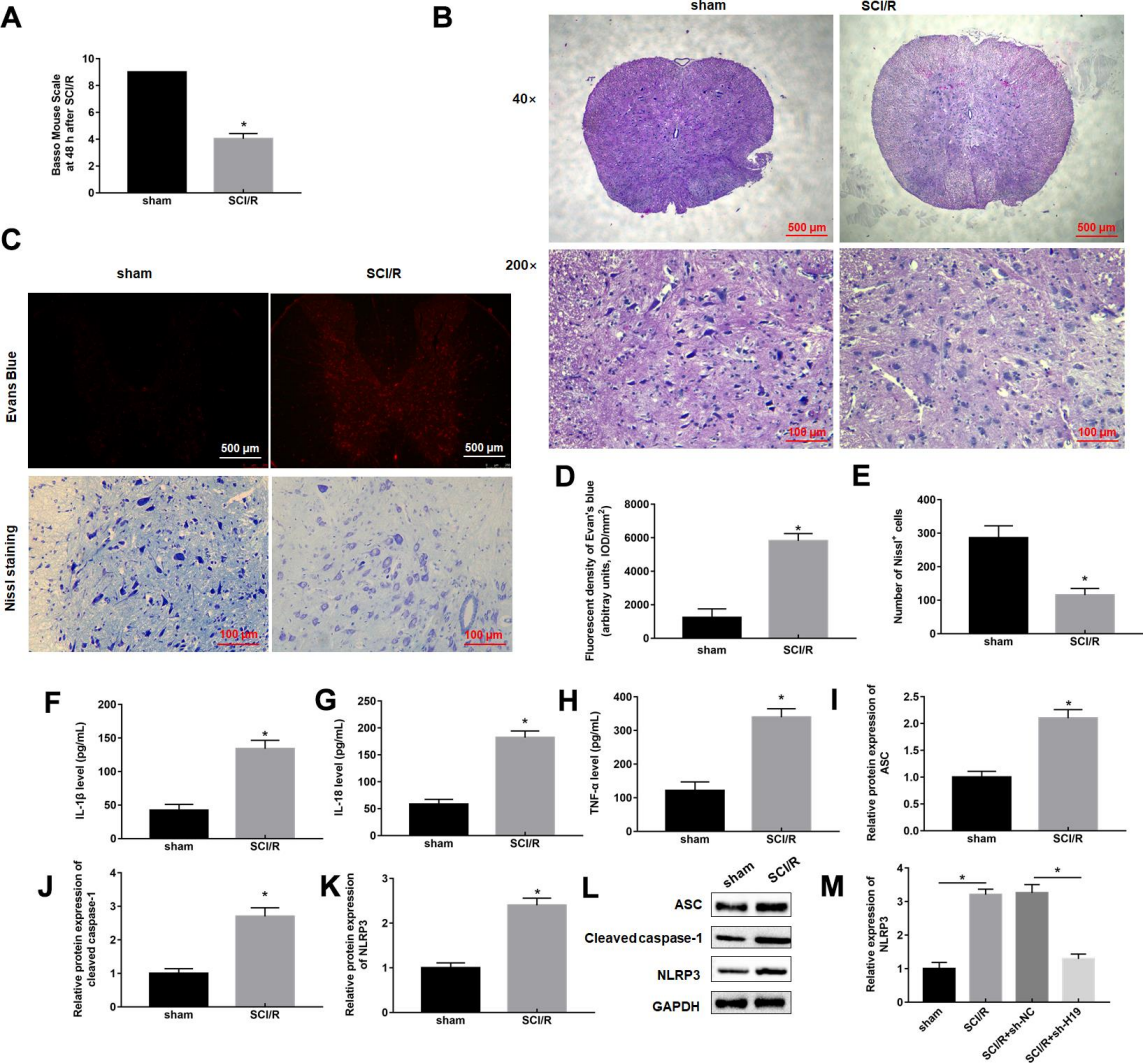
**SCI/R induces pyroptosis and increases H19 expression level.** Mice were subjected to SCI/R or sham surgery. (**A**) The locomotor function of the hind limbs was investigated by the BMS score in mice after SCI/R for 48 h. (**B**) Representative histomorphological changes in the ventral horn at 48 h of reperfusion (HE staining). (**C**) EB fluorescence and neuropathic damage were measured by EB and Nissl staining at 48 h post-injury, respectively. EB (**D**) and Nissl positive cells (**E**) were analyzed quantitatively, respectively. The levels of IL-1β (**F**), IL-18 (**G**), TNF-α (**H**) in serum of mice were determined by ELISA assay. (**I**–**L**) The protein levels of ASC, Cleaved-caspase-1, and NLRP3 were determined by Western blotting. (**M**) The expression levels of H19 in the mouse spinal cord tissues following SCI/R. The results were presented as the mean ± SD. N = 10; *P<0.05.

SCI/R surgery was applied to construct SCI/R injury model *in vivo*. We first measured the BMS scores to evaluate the motion function of mice suffering from SCI/R surgery, the results showed an effective reduction of score after 48 h reperfusion compared with sham group (Figure 1A). Our results from hematoxylin-eosin staining showed that SCI/R mice displayed the presence of shrunken nuclei and extensive vacuolar degeneration, indicating a significant loss of intact neurons (Figure 1B). As shown in Figure 1C, 1D, mice of Sham group displayed almost no EB extravasation, and SCI/R group displayed obvious EB extravasation at 48 h post-surgery. Nissl staining showed that the neuronal cell loss and morphological changes were significantly reduced in SCI/R mice (Figure 1C, 1E). Then, the levels of pyroptosis marker proteins, including IL-1β, IL-18, TNF-α, NLRP3, ASC, and caspase-1 were checked, and the results showed that they were all markedly increased in response to SCI/R surgery (Figure 1F–1L), indicating that pyroptosis occurs during SCI/R. Interestingly, we found that lncRNA H19 was robustly upregulated in spinal cord of SCI/R mice (Figure 1M).

### Knockdown of lncRNA H19 reduced OGD/R-induced pyroptosis

**Figure 2:**
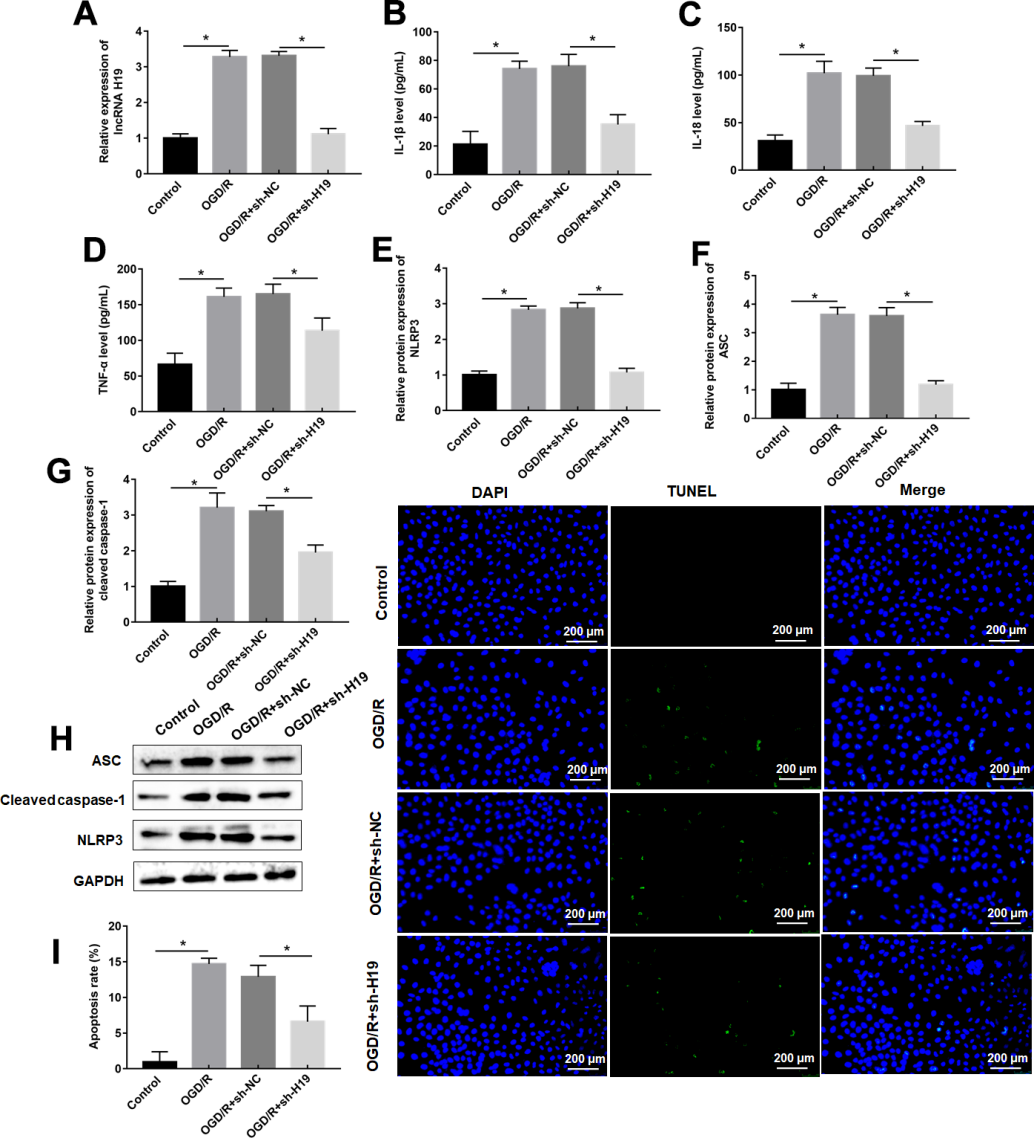
**Knockdown of H19 suppresses OGD/R-induced pyroptosis.** PC12 cells were transfected with sh-H19 or sh-NC and then subjected to OGD/R. (**A**) Relative levels of H19 in transfected PC12 cells following OGD/R exposure. (**B**–**D**) The levels of IL-1β, IL-18, TNF-α were determined in transfected PC12 cells following OGD/R exposure by ELISA. (**E**–**H**) The protein levels of NLRP3, ASC, and Cleaved-caspase-1 were determined in transfected PC12 cells by Western blotting. (**I**) Cell apoptosis rate was identified by TUNEL assay. The results were presented as the mean ± SD. N = 3; *P<0.05.

OGD/R exposure increased H19 expression level in PC12 cells, which was dispelled by sh-H19 transfection (Figure 2A). OGD/R treatment remarkably suppressed expression of proinflammatory proteins and pyroptosis marker proteins, and apoptosis rate in PC12 cells compared to control cells (Figure 2B–2I), which was also inverted by sh-H19 transfection (Figure 2B–2I).

### LncRNA H19 acted as a molecular sponge to inhibit miR-181a-5p

**Figure 3:**
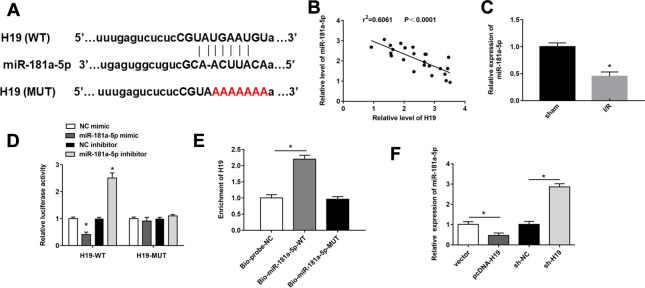
**H19 acted as miR-181a-5p sponge to suppress its expression.** (**A**) The sequences H19 containing the miR-181a-5p binding sites or mutant binding sites were showed. (**B**) The expression of miR-181a-5p was detected by RT-qPCR. (**C**) The correlations of the H19 and miR-181a-5p expression levels in the mouse spinal cord tissues following I/R were analyzed by Pearson correlation analysis. (**D**, **E**) luciferase reporter gene assay and RNA pull-down were used to detect the interaction between miR-181a-5p and H19 in PC12 cells. (**F**) The expression of miR-181a-5p was detected by RT-qPCR. The results were presented as the mean ± SD. N = 3; *P < 0.05.

We used bioinformatic analysis to predict the binding site between H19 and miR-181a-5p (Figure 3A). MiR-181a-5p had been previously proven to be conducive to inhibit the inflammatory process in ischemia-reperfusion injury [25]. miR-181a-5p was downregulated in spinal cord tissues of mice after SCI/R for 48 h after SCI/R injury compared with sham group (Figure 3B). Besides, there was a negative correlation between miR-181a-5p and H19 in SCI/R spinal cord (Figure 3C). miR-181a-5p mimics markedly suppressed luciferase activity in the luci-H19-WT group, whereas miR-181a-5p inhibitor increased that (Figure 3D). Afterwards, RNA-pull down assay revealed that in PC12 cells, H19 was abundant in bio-miR-181a-5p-WT pulled down RNA complex, but not in the bio-miR-181a-5p-MUT or the NC probe (Figure 3E). Furthermore, H19 overexpression successfully reduced miR-181a-5p expression, while H19 silencing significantly increased miR-181a-5p levels (Figure 3F).

### miR-181a-5p attenuates pyroptosis caused by OGD/R

**Figure 4:**
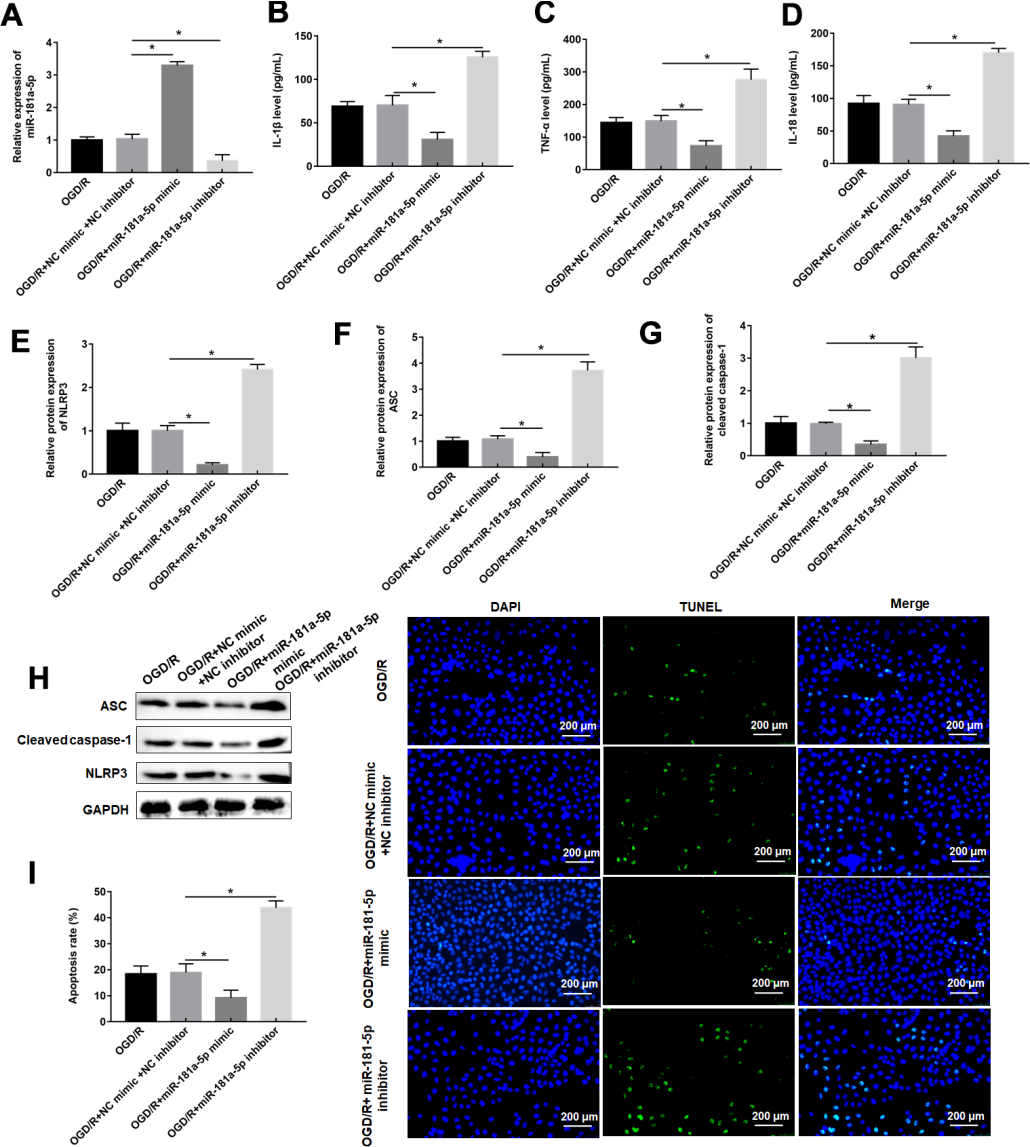
**MiR-181a-5p attenuates pyroptosis caused by OGD/R.** PC12 cells were transfected with miR-181a-5p mimic or miR-181a-5p inhibitor or their negative control and then subjected to OGD/R. (**A**) Relative levels of miR-181a-5p in transfected PC12 cells following OGD/R exposure. (**B**–**D**) The levels of IL-1β, IL-18, TNF-α were determined in transfected PC12 cells following H/R exposure by ELISA. (**E**–**H**) The protein levels of NLRP3, ASC, and caspase-1 were determined in transfected PC12 cells by Western blotting. (**I**) Cell apoptosis rate was identified by TUNEL assay. The results were presented as the mean ± SD. N = 3; *P<0.05.

MiR-181a-5p mimic had a good promoting effect on miR-181a-5p expression, whereas miR-181a-5p inhibitor showed an inhibitory effect in OGD/R-administrated PC12 cells (Figure 4A). In response to miR-181a-5p overexpression, the increases of PC12 cell inflammation and apoptosis induced by OGD/R treatment were reversed, as reflected by suppressed of proinflammatory proteins and pyroptosis marker proteins, as well as decreased apoptosis rate (Figure 4B–4I). miR-181a-5p inhibitor further enhanced these changes in inflammation response and apoptosis resulted from OGD/R treatment (Figure 4B–4I).

### MiR-181a-5p targeted and negatively regulated HMGB1

**Figure 5:**
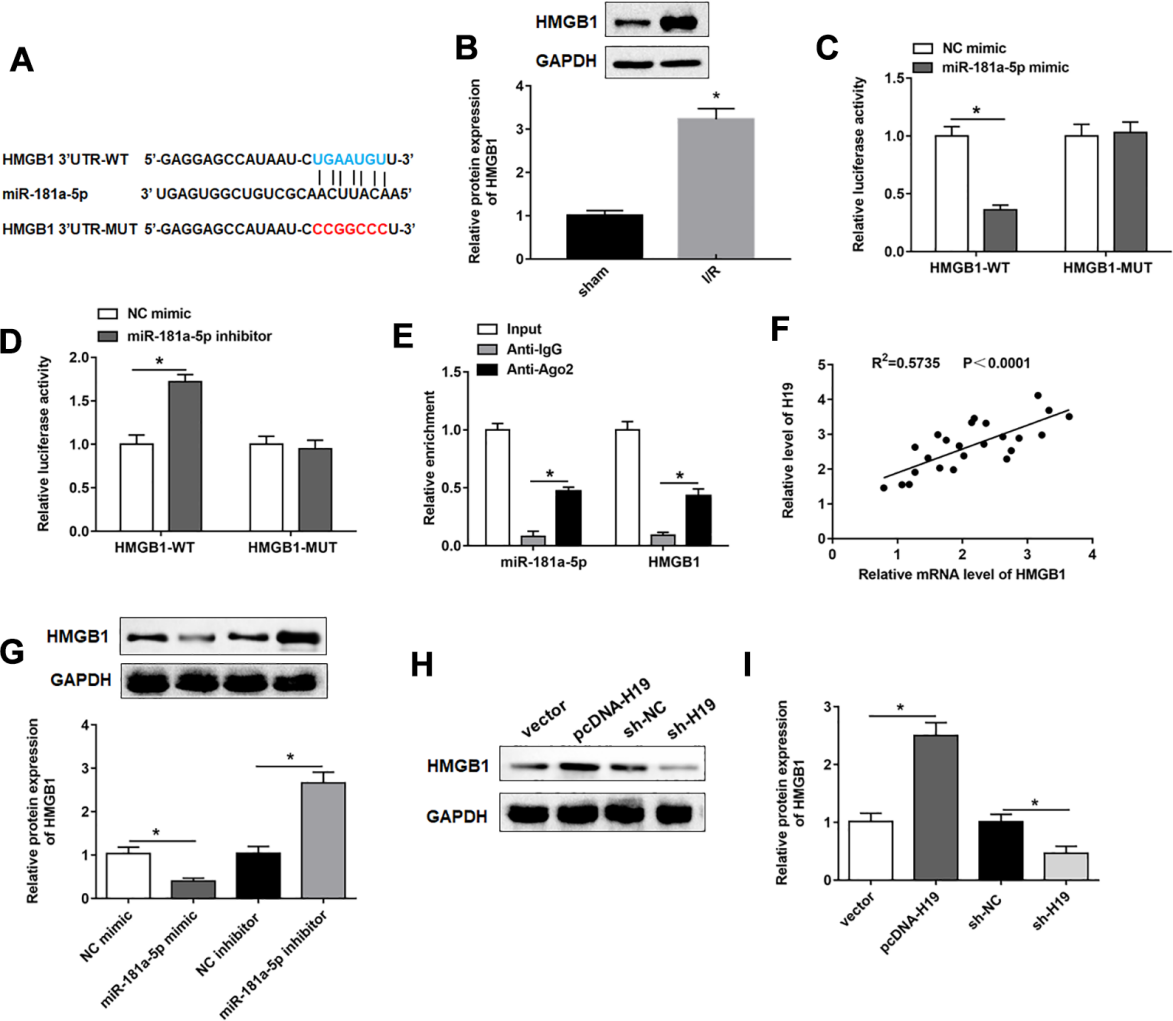
**MiR-181a-5p targeted and negatively regulated HMGB1.** (**A**) The sequences of HMGB1 3ʹUTR containing the miR-181a-5p binding sites or mutant binding sites were showed. (**B**) The expression of HMGB1 in the mouse spinal cord tissues following I/R. (**C**, **D**) Luciferase reporter gene assay was used to detect the luciferase activities of HMGB1-WT and HMGB1-MUT. (**E**) RIP assay was performed to determine the enrichment of miR-181a-5p and HMGB1 in Anti-Ago2 or IgG. (**F**) The correlations of the H19 and HMGB1 expression levels in the mouse spinal cord tissues following I/R were analyzed by Pearson correlation analysis. (**G**) The expression of HMGB1 was examined in PC12 cells transfected with miR-181a-5p mimic, miR-181a-5p inhibitor and their negative controls. (**H**, **I**) The expression of HMGB1 was examined in PC12 cells transfected with pcDNA-H19, sh-H19 and their negative controls. The results were presented as the mean ± SD. N = 3; *P<0.05.

We then used the Starbase database (http://starbase.sysu.edu.cn/index.php) to predict the target genes of miR-181a-5p, and the results showed a putative target site between miR-181a-5p and HMGB1 3’UTR (Figure 5A). It was reported that HMGB1 played an important role in pyroptosis [26]. Consistent with previous data [27], the expression of HMGB1 was upregulated in SCI/R spinal cord (Figure 5B). Their targeting relationship was confirmed with dual luciferase reporter gene assay (Figure 5C, 5D). miR-181a-5p and HMGB1 were enriched in anti-Ago2-precipitated RNA-protein complex, but not in that of anti-IgG (Figure 5E). In addition, there was a positive correlation between H19 and HMGB1 in SCI/R spinal cord (Figure 5F). Overexpression of miR-181a-5p significantly reduced the protein expression of HMGB1, while miR-181a-5p inhibition increased the expression of HMGB1 in PC12 cells (Figure 5G). HMGB1 was sharply down-regulated by sh-H19 transfection, and markedly increased in response to pcDNA-H19 transfection (Figure 5H, 5I).

### LncRNA H19 knockdown inhibited OGD/R-induced pyroptosis by downregulating HMGB1

**Figure 6:**
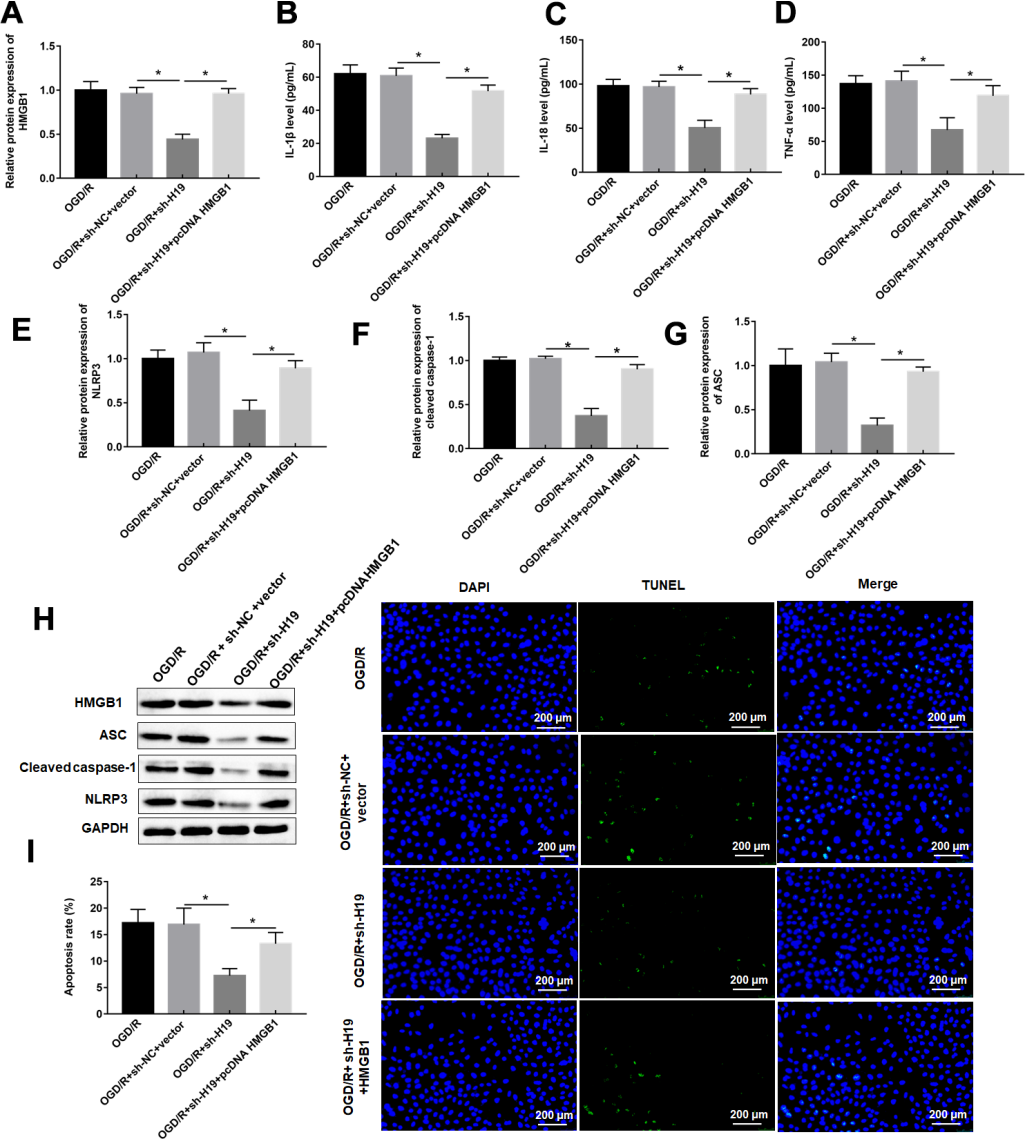
**LncRNA H19 knockdown inhibited OGD/R-induced pyroptosis by downregulating HMGB1.** PC12 cells were transfected with sh-H19 alone or together with pcDNA-HMGB1 and then subjected to OGD/R. (**A**) Relative levels of HMGB1 in transfected PC12 cells following OGD/R exposure. (**B**–**D**) The levels of IL-1β, IL-18, TNF-α were determined in transfected PC12 cells following H/R exposure by ELISA. (**E**–**H**) The protein levels of NLRP3, ASC, and caspase-1 were determined in transfected PC12 cells by Western blotting. (**I**) Cell apoptosis rate was identified by TUNEL assay. The results were presented as the mean ± SD. N = 3; *P<0.05.

Western blotting results showed that overexpression of HMGB1 reversed the inhibitory effect of H19 silencing on HMGB1 expression (Figure 6A). As expected, overexpression of HMGB1 restored the changes of pyroptosis marker protein levels, and apoptosis rate induced by H19 interference via upregulating pyroptosis marker proteins, and pyroptosis rate (Figure 6B–6I).

### LncRNA H19 aggravated spinal cord I/R injury through miR-181a-5p/HMGB1 axis *in vivo*

**Figure 7:**
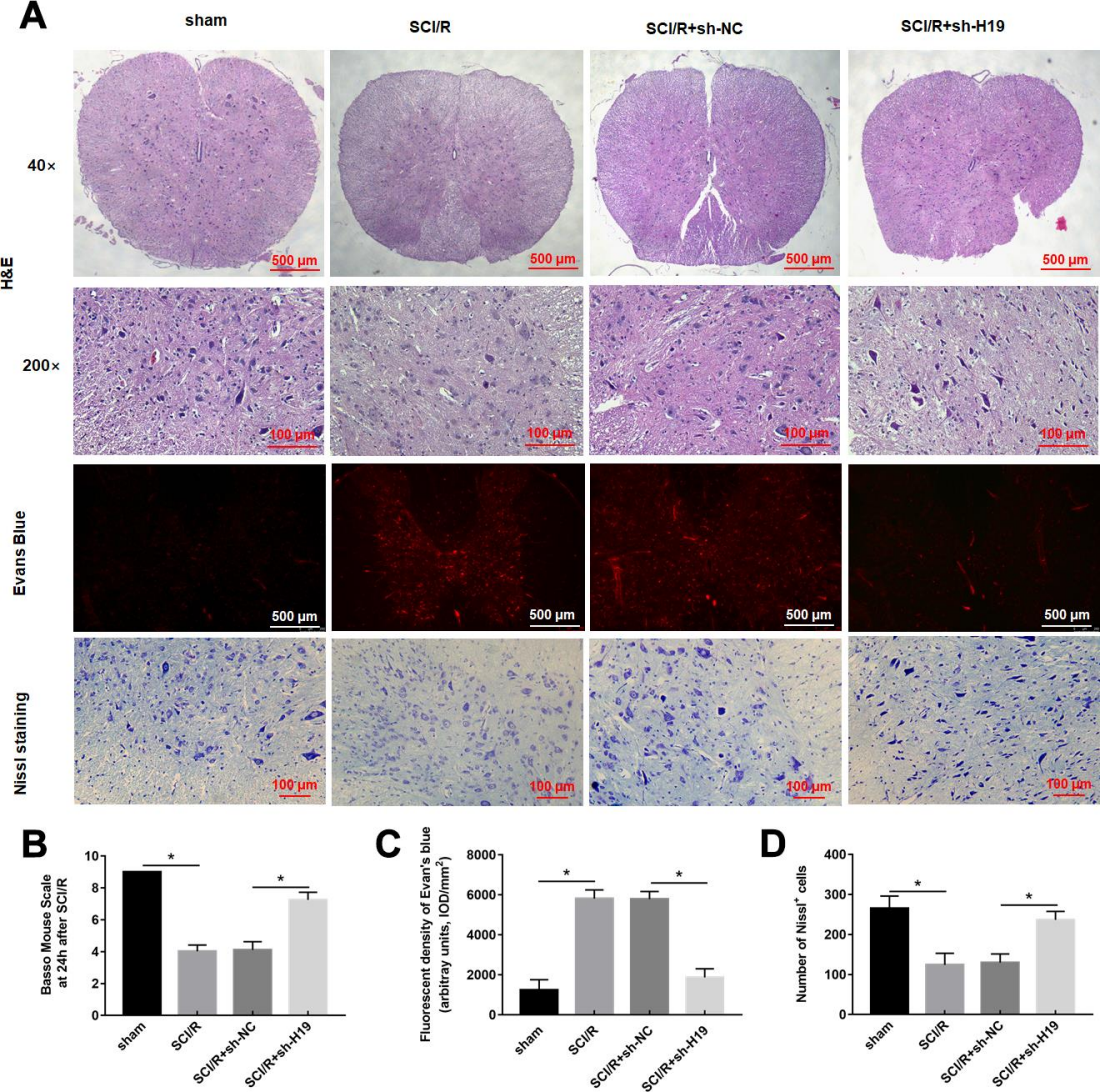
**Downregulation of H19 alleviates SCI/R injury in SCI/R mice.** (**A**) H&E, Evans Blue Extravasation, and Nissl staining (× 200) were investigated in mice after SCI/R for 48 h. (**B**) The locomotor function of the hind limbs was investigated by the BMS score in mice after SCI/R for 48 h. EB (**C**) and Nissl positive cells (**D**) were analyzed quantitatively, respectively. The results were presented as the mean ± SD. N = 10; *P<0.05.

**Figure 8:**
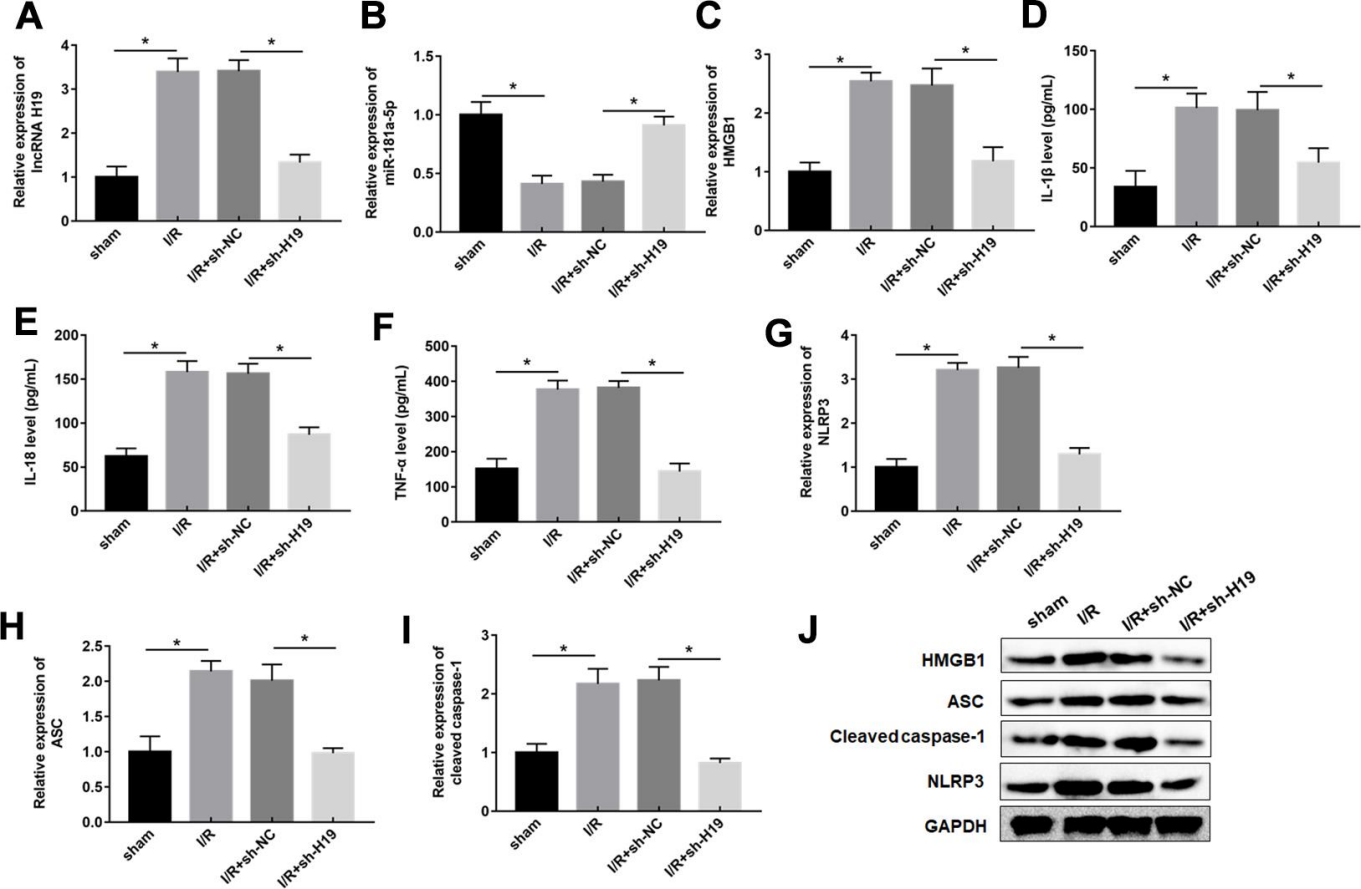
**Downregulation of H19 decreased inflammation response by upregulation of miR-181-5p in SCI/R mice.** (**A**–**C**) The expression levels of H19, miR-181a-5p, and HMGB1 in the spinal cord tissues of mice following SCI/R were determined by RT-qPCR and western blot analysis. (**D**–**F**) The levels of IL-1β, IL-18, TNF-α in serum of mice were determined by ELISA assay. (**G**–**J**) The protein levels of NLRP3, ASC, and caspase-1 were determined by Western blotting. The results were presented as the mean ± SD. N = 10; *P<0.05.

Based on the above-mentioned results, it was speculated that H19 aggravated spinal cord SCI/R injury through miR-181a-5p/HMGB1 axis. Therefore, mice were intrathecally injected with either sh-H19 lentivirus or sh-NC lentivirus after exposure to SCI/R. Nissl staining and HE assays revealed that SCI/R injury caused a significant loss of intact neurons. H19 interference preserved the fine granular cytoplasm of the neurons (Figure 7A, 7D). And the results also showed a significantly increase in the EB extravasation, while H19 silence reversed this effect (Figure 7A, 7C). As shown in Figure 7B, the results revealed that the sh-H19 improved locomotor activity of SCI/R mice. Furthermore, H19 silencing led to the downregulation of H19, up-regulation of miR-181a-5p and down-regulation of HMGB1 protein (Figure 8A–8C). In addition, treatment with sh-H19 significantly inhibited SCI/R-induced neuronal pyroptosis (Figure 8D–8J).

## DISCUSSION

Current investigations indicated that pyroptosis plays a critical role in the pathogenesis of SCI/R [28]. Kim et al. reported that inactivation of NLRP3 and AIM2 inflammasomes attenuated I/R-induced hepatic injury [29]. However, detailed underlying mechanism of inflammation process in SCI/R has not been clarified. AIM2 inflammasome contributed to spinal cord injury by releasing cleaved caspase-1 and IL-1β [9]. The expression of HSPA8 was efficiently enhanced after SCI/R injury, which activated the NF-κB-NLRP3 inflammasome signaling, resulting in spinal ischemia-reperfusion injury [30]. In this study, we demonstrated that OGD/R treatment promoted PC12 cell pyroptosis and inflammation, while knockdown of H19 significantly reversed these results, indicating H19 protect PC12 cells from OGD/R [31].

Previous studies revealed that lncRNA H19 was significantly increased, and suppressing H19 alleviated the effects of OGD or LPS treatment on cell inflammation and pyroptosis [32]. Moreover, lncRNA H19 silence inhibited LPS-induced astrocyte activation post SCI/R, where H19 weakened miR-1-3p-induced inhibition of CCL2 expression by acting as a ceRNA [33]. Herein, we demonstrated that H19 directly interacted with miR-181a-5p to cut down its function in neuronal cells, which was consistent with a previous report [34].

MiRNAs are important regulators in SCI/R. For example, miR-214 expression was increased in the rat spinal cord tissues and hypoxia/reoxygenation (H/R) H/R-treated PC12 cells, and miR-214 overexpression inhibited cell viability and enhanced cell apoptosis in PC12 cells after exposed to H/R, contributing to the exacerbation of SCI/R injury [35]. Wang et al. found that miR-30c decreased Basso, Beattie and Bresnahan (BBB) score and promoted apoptosis and inflammation by inhibiting SIRT1 expression, thus aggravating SCI/R injury [36]. Cochlear spiral ganglion progenitor cell-derived exosomes could protect cochleae damage from SCI/R through upregulating miR-181a-5p via inhibiting the inflammatory process [25]. Song et al. revealed that miR-181a-5p was highly expressed in serum of acute I/R patients, mice, and OGD/R-induced N2a cells, which targeted En2 to block the Wnt pathway [37]. A study showed that miR-181a-5p alleviated neuronal injury in Parkinson’s disease cell model by inhibiting CXCL12 [16]. In our study, miR-181a-5p was poorly expressed in SCI/R spinal cord in mice. Our data firstly demonstrated that miR-181a-5p alleviated pyroptosis and pyroptosis-regulated programmed cell death in PC12 cells after OGD/R treatment, as evidenced by the reduction of pyroptosis marker genes and PI positive cell ratio.

Previous studies showed that miR-181a-5p interacted with HMGB1 to suppress the expression of HMGB1, thus participating in the procession of several diseases [38, 39]. Consistently, HMGB1 was upregulated in the spinal cord tissues of mice exposed to SCI/R and miR-181a-5p negatively regulated expression of HMGB1, which suggested that HMGB1 showed an opposite expression pattern compared with miR-181a-5p. More importantly, increasing evidence has reported the important roles of HMGB1 in SCI/R injury. Liu et al. revealed that SCI/R increased the expression of HMGB1, Dexmedetomidine inhibited the inflammatory response and stabilize the integrity of blood-spinal cord barrier via blockade of the HMGB1-TLR4-NF-κB signaling pathway [40]. Li et al. showed that miR-129-5p overexpression protected against IR by inhibiting HMGB1 and TLR3-associated cytokines to repress neuron inflammation [41]. In addition, a study found that Glycyrrhizin attenuated hepatic I/R injury by suppressing HMGB1-dependent GSDMD-mediated Kupffer cells pyroptosis [26], and HMGB1 contributed to inflammation response by activating the NLRP3 inflammasome and thereby initiating cell pyroptosis [42]. Here, we demonstrated that knockdown of H19 inhibited pyroptosis and pyroptosis-mediated cell death via downregulating HMGB1 expression through increasing the expression of miR-181a-5p.

In conclusion, lncRNA H19/miR-181a-5p/HMGB1 ceRNA network contributes to spinal cord ischemia/reperfusion injury through increasing neuronal pyroptosis by miR-181a-5p/HMGB1 axis, which may be a new therapeutic target for SCI/R injury.
